# 

**Published:** 1880-04

**Authors:** 


**TERMS.—50 Cents a Year. Address The Bistoury, Elmira, N. Y.**

### SUBSCRIPTIONS RECEIVED TO APRIL, 1880.

In this column we will credit every subscription received during the last quarter. Subscribers will please notify us immediately if their payments are not duly credited. The State only is given—this, with the full name of the party subscribing, will be sufficient to indicate who is meant.

NEW YORK—Dr S M Prentice $1.00, A B Abbott, Bundy Bros, H B Berry, Dr S G Ellis, H R Rogers, E T Walker, Jesse Owen, T W Elmore, W F Wentz, J B Parkinson, J B Ells, Wm Nuttall, Dr D Everett, H L Bacon $1.10, A Houghton Jr, Dr M T Babcock, H S Gilbert, C L Harrington $2.00, Dr P S Smith, Clinton Smith, Jno Woolworth, F M Jones, Dr H A Bolles, Dr O C Clark $1.00, A Tillotson, G H Metcalfe, S H Ainsworth, Geo P Johnson, C V Bush, H B Gilbert, M S Palmer, Col. J H Horton, Wm Collins, Newton Graves, Dr H D Welles, Dr Geo R Kinney, Mrs M L Keeney $1.00, Dr A Osgood $2.00, M J Wilcox $1.50, Dr N R Seeley, Dr J C Dixon, L E Hewitt, Mrs J C Seeley, Dr C S Carr, Mrs J M Preston, Dr S A Cottrell, Dr H M Paine Dr Ira D Hopkins, L E Goulding, *Practical American*, Dr Walter Booth, W O Osbourne, G B Graves, Mrs J F Dayton, Dr W H Jackson, Mrs P A LaFrance, Dr R J Stewart, Miller Bros, *American Jour. Microscopy*, Chas E Stagg. Ingraham Bros, F G Hall, Dr Leonard, S S Hamlin, W J Lormore, G P Rawson, Dr L D Parkhurst, N P Fassett, E B Youmans. Geo Dorn, Dr Darby, L A Humphrey.

PENNSYLVANIA—S R Stigben, Dr. J R Fordham, Prof A S Low, J B Chandler. W H Nicholas, R S Wallace, Drs. W H & G M Romig, R F Potter, J H McMinn, Dr. J N Wright, Dr J R Shellenberger, Dr I W Meare $1.00, Dr S B Hartman $1.00, Dr D P Miller $3.00, G H Platt, W C Richardson, J C Wollison, W C Hyde $1.00, Alex Beede Dr Max J Reinhold $1.00, R J Wisner, Robt Innes, R Smith.

ALABAMA—A A Woodward, J Olin Zeigler, Dr G W Files, Dr S M Emmett $4.00, Dr O N Oglisbie, Dr Rich'd Chase, Dr E D Phillips, Dr C P Preautaugh.

ARKANSAS—Dr J R Hawkins, Wm H Beede, Dr Saml Johnston $3.00, Hiram Potter, Mrs L B Otto, Drs Ellwanger & Bishop, Dr D L Frye.

CALIFORNIA—Dr J F Gibbon, Dr G L Simmons, Wm S Burns $1.00, Dr A B Conkling $3.00, Dr D D Bishop $2.00, Dr Henry English $2.00, Dr A L Gemberling $2.00, Dr S B Hines $1.00, L L Dufor $1.00.

CONNECTICUT—Mrs M A Bradford $1.00, Dr Garry H Minn $2.00, Dr D B Mercereau $4.00, D V H Art $2.00, Dr Sam Fricke $2.00, Dr Harris Burns,

DISTRICT OF COLUMBIA—M D Reiling $2.00, Dr D O Hardin, Dr Emberling.

FLORIDA—Dr E T Sabal, Dr A J Wakefield $1.00, Dr R B Burroughs, Calvin Oak, Dr N C Stevens, Dr J D Hinds $3.00, Dr R M Frothingham $2.00, Dr L B Jones $1.00, Dr A N Fowler $1.00.

GEORGIA—Dr C S Strother, Dr Albert Loomis $2.00, Dr H H Henderson $1.00.

INDIANA—Dr J W Compton, C H High'ey, Dr T C Kimball, Dr H D Kingsley $4.00, Dr Davis Young $1.00, Drs Smith & Richardson $3.00, Dr O D Pillsbury $1.00. Dr DeWitt Struther $2.00.

ILLINOIS—Dr W H H Crane, Dr W Bradley, Mrs L D Holley, Dr Dox D Weyburn $3.00, Drs Henderson & Bradley $2.00.

IOWA—Hatton & Young, Dr D Scofield $1.00.

KENTUCKY—Dr Geo L Compton, Dr S S Stickland $3.00, Dr Strouthers $2.00.

LOUISIANNA—Dr L R Meretsick $2.00, Dr Delano $1.00.

MASSACHUSETTS—Miss Howland, Thos H Manley, Dr E P Hurd, A Wood, Dr P Snow $3.00, Dr Hiram Butler $2.00, Dr A Kingsbury $1.00, Dr Emberlie $1.00.

MICHIGAN—Dr E C Davis, Mrs C J Davis, Dr J W Babbitt $1.00, Dr Harvey Loomis, Dr A Garwood, Dr V A Baker $1.00.

MARYLAND—Dr Jno S Aydelotte, Dr Steve Angell $2.00, Dr H Emerson $2.00.

MINNESOTA—Mrs A W Welles, J W Foss.

MISSISSIPPI—Dr J C Robert, Dr Jos Redhead, W C Sillman $1.00, Dr Geo D Prentice $2.00.

NEW HAMPSHIRE—Edward Runnels $1.00, Dr S M Emery, Dr Floyd Stamp $3.00, Dr D V Hurling $2.00.

NEW JERSEY—Dr Boardman Reed, Jas F Forwood, J M Atwater.

OHIO—C H Comstock, T Bradley, Dr F S Hillbish $1.00.

SOUTH CAROLINA—Dr John W Ogelvie $1.50, Dr. D. L. Anderson.

TENNESSEE—Drs J P & W C Dake, Dr A P Grinstead.

TEXAS—Dr John Hoffman $1.00, W. D. Hoskins $1.00, Dr Wm H Holt $1.00, Dr Jas Cowling.

VERMONT—Chas T Barrett, T A Kingsley, Dr G S Simpson $3.00. Dr D A York $1.00, Dr S S Sanders $2.00.

WISCONSIN—G W Sheardown $1.00, Dr. S. B. Hart $3.00, Dr Nat Ralph, Drs Hunt & Frye $2.00.

NO NAME—$2.00 from Jackson, Miss.

NO PLACE—Dr O M Hartz $2.00.

